# Inflammation induced by mast cell deficiency rather than the loss of interstitial cells of Cajal causes smooth muscle dysfunction in *W/W^v^* mice

**DOI:** 10.3389/fphys.2014.00022

**Published:** 2014-02-04

**Authors:** John H. Winston, Jinghong Chen, Xuan-Zheng Shi, Sushil K. Sarna

**Affiliations:** Division of Gastroenterology, Department of Internal Medicine, Enteric Neuromuscular Disorders and Visceral Pain Center, The University of Texas Medical Branch at GalvestonGalveston, TX, USA

**Keywords:** enteric nervous system, motility disorders, ICC, nNOS, enteric neurotransmission, nitric oxide, diabetes, slow transit constipation

## Abstract

The initial hypothesis suggested that the interstitial cells of Cajal (ICC) played an essential role in mediating enteric neuronal input to smooth muscle cells. Much information for this hypothesis came from studies in *W/W^v^* mice lacking ICC. However, mast cells, which play critical roles in regulating inflammation in their microenvironment, are also absent in *W/W^v^* mice. We tested the hypothesis that the depletion of mast cells in *W/W^v^* mice generates inflammation in fundus muscularis externa (ME) that impairs smooth muscle reactivity to Ach, independent of the depletion of ICC. We performed experiments on the fundus ME from wild type (WT) and *W/W^v^* mice before and after reconstitution of mast cells by bone marrow transplant. We found that mast cell deficiency in *W/W^v^* mice significantly increased COX-2 and iNOS expression and decreased smooth muscle reactivity to Ach. Mast cell reconstitution or concurrent blockade of COX-2 and iNOS restored smooth muscle contractility without affecting the suppression of c-kit in *W/W^v^* mice. The expression of nNOS and ChAT were suppressed in *W/W^v^* mice; mast cell reconstitution did not restore them. We conclude that innate inflammation induced by mast cell deficiency in *W/W^v^* mice impairs smooth muscle contractility independent of ICC deficiency. The impairment of smooth muscle contractility and the suppression of the enzymes regulating the synthesis of Ach and NO in *W/W^v^* mice need to be considered in evaluating the role of ICC in regulating smooth muscle and enteric neuronal function in *W/W^v^* mice.

## Introduction

*c-kit* proto-oncogene encodes type III receptor tyrosine kinase (kit) (Chabot et al., 1988; Geissler et al., 1988). Stem cell factor (SCF) is the ligand for kit (Williams et al., 1990). The activation of kit regulates important cellular functions, including proliferation, migration, apoptosis, chemotaxis and adhesion that are critical for the development and maintenance of several cell types—hematopoietic cells, mast cells, melanocytes, gametocytes, and interstitial cells of Cajal (ICC) (Kitamura and Go, 1979; Huizinga et al., 1995; Bernex et al., 1996; Wu et al., 2000). The non-lethal point mutations in *c-kit* in mice (*W/W^v^* mice) have served well to investigate macrocytic anemia (Till et al., 1967), decrease in fertility (Kissel et al., 2000), pigmentation (Poole and Silvers, 1979), and mast cell deficiency (Piconese et al., 2011).

In mid-nineties, the *W/W^v^* mice lacking ICC in the gut wall were adopted to investigate the role of ICC in regulating motor function (Burns et al., 1996). Since then, several *in vitro* studies reported that smooth muscle relaxation in response to electrical field stimulation (EFS) and slow waves were absent in *W/W^v^* mice. Based on these findings, the investigators proposed that the ICC played an essential role in mediating enteric neuronal input to smooth muscle cells (Burns et al., 1996; Ward et al., 1998, 2000; Wang et al., 1999; Suzuki et al., 2003). The tacit assumption in arriving at this conclusion was that the observed changes in gut motor function or its regulatory mechanisms in *W/W^v^* mice were only due to the deficiency of ICC. However, the *W/W^v^* mice are also severely deficient in mast cells (Wershil and Galli, 1994). Mast cells are innate immune cells; in response to antigen challenge, they release proinflammatory mediators that potentiates effector cell recruitment and complements other components of the immune system to enhance the inflammatory response (Mekori and Metcalfe, 1999; Galli et al., 2008). Mast cells also have an anti-inflammatory function (Galli et al., 2008). Other effects of mucosal mast cell deficiency in W-sh/W-sh mice are, increase in permeability of intestinal segments by increased crypt depth, decrease in migration of epithelial cells and down regulation of expression of the tight junction protein claudin-3 (Groschwitz et al., 2009). The decrease of claudin-3 impairs tight junction integrity (Milatz et al., 2010) that effectively allows increase in penetration of luminal pathogens. On the other hand, mast cell deficiency in IL-10^−/−^ mice predisposes them to the development of spontaneous colitis (Chichlowski et al., 2010). The effects of mast cell deficiency on smooth muscle and enteric neurons in *W/W^v^* mice remain unknown.

We tested the hypothesis that mast cell deficiency in *W/W^v^* mice generates an inflammatory environment in the muscularis externa of the fundus that impairs smooth muscle contractility to Ach and the expression of ChAT and nNOS, the two enzymes regulating the synthesis of Ach and NO, respectively. We found that the *W/W^v^*mice had elevated levels of iNOS and COX-2 in the fundus muscularis externa that suppressed smooth muscle reactivity to Ach. The expression of c-kit was reduced and ICC networks were absent in *W/W^v^* mice, as expected. Reconstitution of mast cells by bone marrow transplant (Grimbaldeston et al., 2005) reduced iNOS and COX-2 levels, and reversed the suppression of smooth muscle reactivity to Ach, without affecting the impaired c-kit expression and ICC networks. Our findings establish a cause-and-effect relationship between inflammation induced by mast cell deficiency and impaired smooth muscle function in *W/W^v^*mice. ChAT and nNOS expression were also suppressed in the muscularis externa of the *W/W^v^* mice; mast cell reconstitution did not restore their expression to wild type levels. Our findings suggest that the absence of mast cells in *W/W^v^*mice generates an inflammatory environment that impairs smooth muscle function and the expression of ChAT and nNOS independent of depletion of ICC. These effects should be considered in evaluating the putative roles of ICC in regulating motility function in *W/W^v^*mice.

## Materials and methods

### Mice

WBB6F1/J-KitW/KitW-v mice and control WBB6F1/J−+/+ littermates were purchased from Jackson Laboratories. The UTMB Institutional Animal Care and Use Committee approved all manipulations performed on mice in this study.

### Mast cell reconstitution

Bone marrow was isolated from the hind leg femur and tibia of WBB6F1/J−+/+ littermates in Hanks balanced salt solution on ice. Cell number was determined by counting aliquots on a hemacytometer; 1 × 10^7^ cells in 0.1 ml sterile saline were injected into the tail vein of *W/W^v^* mice; controls received saline. Mast cell reconstituted *W/W^v^*mice and control *W/W^v^* and wild type (WT) littermates remained in the vivarium for 3 months before using them for experiments. Mast cell reconstitution by bone marrow transplant is an established procedure (Grimbaldeston et al., 2005); however, we cannot rule out a change in mast cell phenotype during reconstitution.

### Tissue preparation

Mice were euthanized by CO_2_ inhalation, and the stomachs were removed, cut open along the greater curvature, cleansed with Kreb's buffer (2.5 mM CaCl_2_· 2H_2_O, 5.5 mM KCl, 1.2 mM MgCl_2._6H_2_O, 15.4 mM NaHCO_3_, 1.5 mM NaH_2_PO_4_, 120.3 mM NaCl, 11.5 mM glucose) and pinned flat serosal side down in Krebs buffer in a sylgard dish. Antrum and fundus were separated and processed for immune studies or snap frozen in liquid nitrogen for molecular studies or prepared for organ bath.

### Smooth muscle reactivity to Ach

Gastric fundus was isolated from the mouse, and immersed in carbonated Krebs buffer (118 mmol/L NaCl, 4.7 mmol/L KCl, 2.5 mmol/L CaC_2_, 1 mmol/L NaH_2_PO_4_, 1.2 mmol/L MgCl_2_, 11 mmol/L D-glucose, and 25 mmol/L NaHCO_3_) and mucosa was removed. The muscle strips (2 mm × 8 mm) were cut along the long axis of the circular muscle and mounted in individual muscle baths (Radnoti Glass, Monrovia, CA) filled with 5 mL carbogenated Krebs buffer at 37°C. Contractile activity was recorded with Grass isometric force transducers (Grass instruments, Quincy, MA) and amplifiers connected to a Biopac data acquisition system (Goleta, CA). The muscle strips were equilibrated in muscle bath under 1 g tension for 60 min before they were tested for contractility. The bath solution was replaced every 15 min. Contractile responses to increasing concentrations of Ach were obtained. The strips were allowed to equilibrate for at least 15 min before adding the next concentration of Ach. The contractile response was quantified as the increase of area under contractions during 4 min after addition of each concentration of Ach over the baseline area under contractions during 4 min before the addition of Ach to the bath. All data were normalized to the response with 10^−6^ M concentration of Ach in wild type mice. At least 2 muscle strips from the fundus of each mouse were used.

### Immunofluorescence staining for c-kit

The ICC were stained for c-kit, as described previously (Horvath et al., 2005). Fundus was pinned flat and fixed with 100% acetone at room temperature for 30 min. After washing twice with water, the tissue was gently shaken in 1 × PBS overnight at 4°C. Next, the tissue was incubated in PBS with 1% BSA and 0.3% Triton X-100 for 2 h at room temperature with gentle shaking and then washed twice with PBS plus 1% BSA for 20 min. Tissue was incubated for 48–72 h at 4°C with gentle shaking in anti-c-kit ACK2 antibody (1:20 in PBS, 1% BSA). The tissue specimen was washed 6 times for 7 min each in PBS plus 1% BSA at room temperature on the shaker. Alexa fluor 568 goat anti-rat IgG H+L (2 mg/ml Invitrogen, Carlsbad, CA) was used as the secondary antibody (1:250 in PBS, 1% BSA). After incubation for 2 h at room temperature, the tissue specimens were washed 5 times and mounted on slides with Fluorosave for fluorescence imaging by an Olympus BX51 microscope powered by DP Controller imaging system.

#### Mast cell staining

Mast cells were visualized by staining formalin fixed paraffin embedded 10 μm fundus sections with Toluidine Blue O. Deparafinized sections equilibrated in water were stained for 3 min in 0.09% sodium chloride, 7% ethanol, 0.1% w/v toluidine O (Sigma Aldrich, St Louis, MO). Sections were washed briefly in water, ethanol, and xylene. Mast cells appeared purple against a light blue background.

### Protein extraction and western blot

For protein extraction, stomach tissue was homogenized on ice in lysis buffer supplemented with protease inhibitors (Sigma-Aldrich, St. Louis, MO). The lysis buffer consists of (in mmol/l) 20 Tris HCl, pH 7.5, 150 NaCl, 1 EDTA, 1 ethylene glycol-bis (β-aminoethyl ether)-*N*,*N*,*N*',*N*'-tetraacetic acid, 2.5 sodium pyrophosphate, 1 β-glycerolphosphate, 1 Na_3_VO_4_, and 1% Triton X-100. Proteins were resolved by standard western blotting, as described previously (Choudhury et al., 2009). Equal quantities (20 μg) of total protein were loaded and run on premade 8–16% Tris-glycine SDS-PAGE (Invitrogen). They were transferred to nitrocellulose membranes (Invitrogen) for incubation with primary and secondary antibodies. Primary antibody to COX-2 (1:1000), obtained from Cayman Chemical (Ann Arbor, MI), recognized a single band at 75 kDa. Anti-β-actin antibody (1:5000, Sigma, St. Louis, MO) recognized a single band at 42 kDa. Anti-iNOS antibody, obtained from Santa Cruz Biotechnology (iNOS C-11, sc-7271), recognized a single 120 kDa band. Anti-c-kit antibody, purchased from Santa Cruz Biotechnology (c-kit C-19, sc-168), consistently recognized a 145 kDa band on blots. Goat anti-ChAT antibody, (1/200 Millipore, Bedford, MA) recognized a single 70 kDa band. Rabbit anti-nNOS antibody [1/200 (Millipore)] recognized a single 155 kDa band. Anti-Heme oxygenase 1 antibody (Santa Cruz Biotechnology, H-105, sc-10789) recognized a single 32 kDa band. IRDye 800-conjugated anti-mouse IgG (Rockland, Gilbertsville, PA) and Alexa Fluor 680 goat anti-rabbit IgG (Invitrogen) were used as secondary antibodies. β-actin band was used as loading control. Detection and analysis were done by Odyssey Infrared Imaging System (LI-COR Biosciences, Lincoln, NE).

### Statistical analysis

Statistical analysis was performed using SigmaPlot version 11.0 (Systat software Inc, USA) or IBM SPSS Statistics 21. All data are expressed as mean ± s.e.m. Muscle bath data derived from drug treated mice were analyzed by Three-Way ANOVA with acetylcholine concentration as a repeated measure, genotype and drug as the two between group factors. Muscle bath data derived from mast cell reconstituted mice were analyzed by Two-Way repeated measures ANOVA with acetylcholine concentration as the repeated measure and mouse type as the between factor. Western blots data were analyzed by Two-Way ANOVA. Fisher's LSD *post-hoc* analysis was used where appropriate. A *p* value < 0.05 was considered statistically significant.

## Results

### Smooth muscle reactivity to Ach and c-kit expression in wild type, *W/W^v^* and mast cell reconstituted *W/W^v^* (*W/W^v^* + MCR) mice

**Figure 1:**
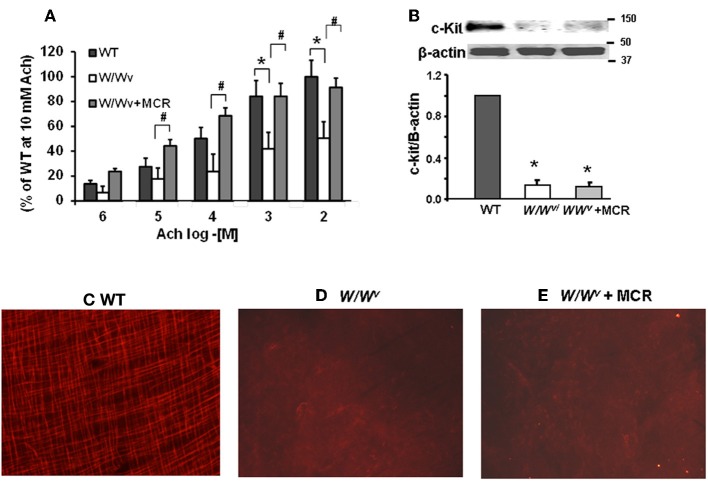
**Smooth muscle reactivity to Ach and c-kit expression in wild-type (WT), *W/W^v^* and *W/W^v^* mast cell reconstituted mice. (A)** Bar graphs displaying the contractile response of fundus circular smooth muscle strips to increasing concentrations of Ach in wild type, *W/W^v^*, and *W/W^v^* with mast cell reconstituted mice. Smooth muscle reactivity to Ach was significantly lower in *W/W^v^* mice vs. the wild type mice (^*^p < 0.05) at Ach concentrations of 10^−3^ and 10^−2^. Mast cell reconstitution restored smooth muscle reactivity to Ach. Smooth muscle reactivity to Ach was significantly higher in *W/W^v^* MCR mice vs. *W/W^v^* mice at Ach concentrations of 10^−5^, 10^−4^, 10^−3^, and 10^−2^ (^#^*p* < 0.05). *N* = 4 mice (7–9 strips) in each group. **(B)** Western blotting showed decrease in expression of c-kit in *W/W^v^*vs. wild type mice that was not restored by mast cell reconstitution. c-kit immunofluorescence in fundus whole mounts from wild type mice showing the network of intramuscular ICC. **(C)** *W/W^v^* **(D)**, and *W/W^v^* + mast cell reconstituted mice **(E)** No intramuscular ICC networks were detected in *W/W^v^* mice. Mast cell reconstitution failed to restore c-kit positive ICC networks.

We measured contractility of fundus circular muscle strips from WT, *W/W^v^* and mast cell reconstituted (MCR) *W/W^v^* mice to investigate whether smooth muscle reactivity to Ach (10^−6^ M to 10^−2^ M) was impaired in *W/W^v^* mice and rescued by mast cell reconstitution. We found significant main effects of mouse type [*F*_2, 100_ = 20.4, *p* < 0.001] and acetylcholine [*F*_4, 100_ = 23.1, *p* < 0.001]. These effects were not dependent upon a significant interaction between mouse type and Ach concentration [*F*_8, 100_ = 1.1, *p* = 0.37]. *Post-hoc* analysis showed that the reactivity of fundus circular smooth muscle strips to Ach in *W/W^v^* mice was significantly lower vs. the wild type mice; mast cell reconstitution completely restored this deficit (Figure 1A).

Western blotting confirmed reduced expression of c-kit in the muscularis externa of the gastric fundus (Figure 1B). Immunostaining with c-kit antibody showed ICC networks in the fundus muscularis externa of the wild type mice (Figure 1C); these networks were absent in *W/W^v^* mice (Figure 1D). Mast cell reconstitution completely reversed the suppression of smooth muscle reactivity to Ach in *W/W^v^* mice (Figure 1A) without affecting the suppressed c-kit expression (Figure 1B) or ICC networks (Figure 1E).

**Figure 2:**
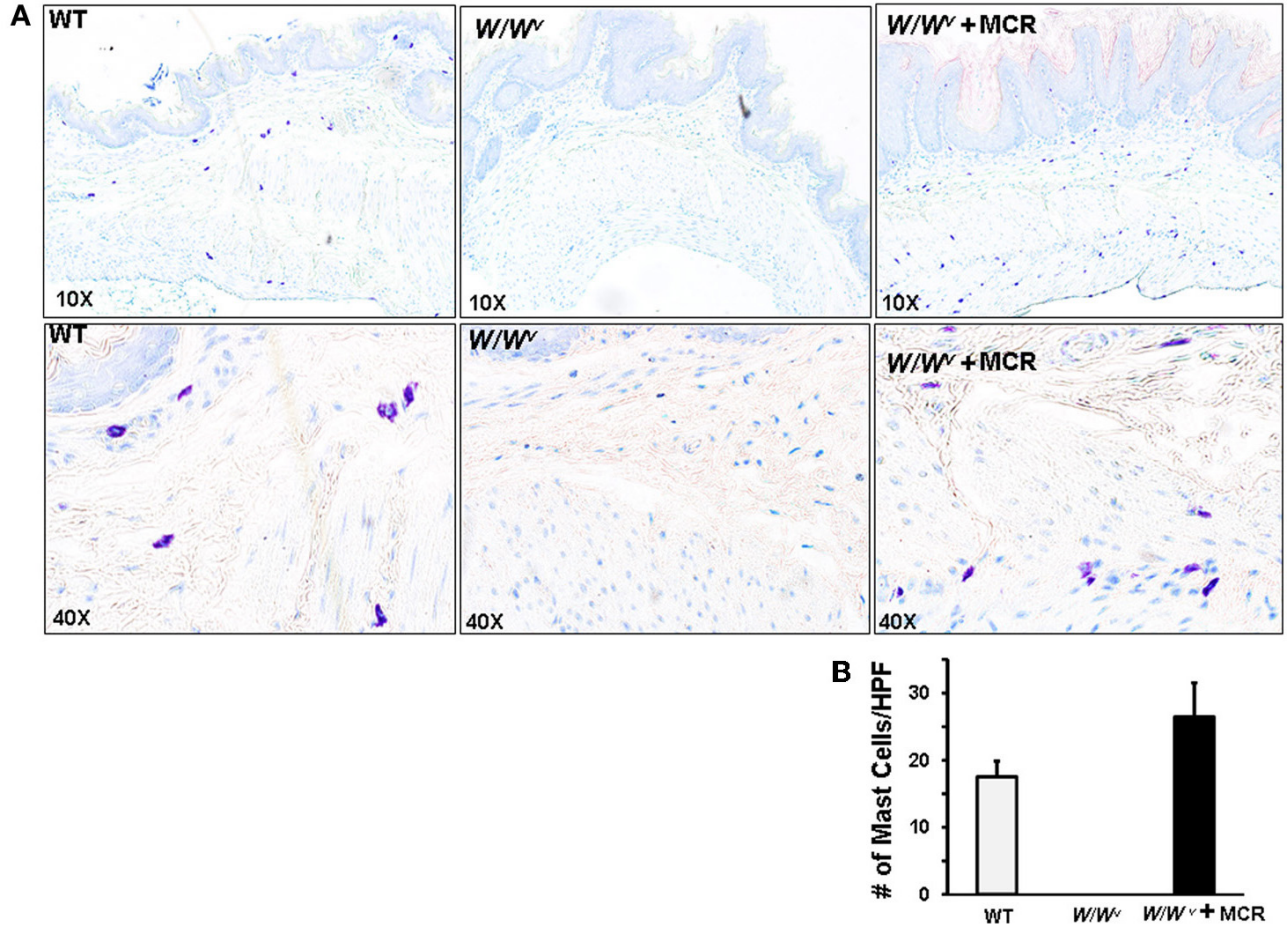
**Mast cell counts in fundus of WT, *W/W^v^* and *W/W^v^* + mast cell reconstitution (MCR). (A)** Panels show formalin fixed paraffin embedded cross sections of fundus tissue stained with the mast cell stain toluidine O. Mast cells appear purple. Top panels are at 10X magnification and bottom panels are 40X magnification. **(B)** Bar graph displays average numbers of mast cell counts per high powered field from three nonserial sections from each mouse, *n* = 5 mice per group. No mast cells were detected in *W/W^v^* sections. Mast cell counts in WT and *W/W^v^* + MCR were not significantly different.

The mast cells in the gastric fundus of the wild type and *W/W^v^* + MCR were distributed in the muscularis externa and mucosa/submucosa (Figure 2). Mast cell distribution observed visually and their numbers/field did not differ between the muscularis externa (9.5 ± 1.7 in WT and 11.5 ± 3.4 in *W/W^v^* + MCR mice *p* > 0.05) and mucosa/submucosa (11.0 ± 2.5 in WT and 13.0 ± 2.5 in *W/W^v^* + MCR mice). The combined total density of reconstituted mast cells in the gastric fundus of the *W/W^v^* mice was not significantly different from that in the wild type mice (Figure 2B).

### Effect of mast cell depletion and reconstitution on the inflammatory environment in the fundus muscularis externa

**Figure 3:**
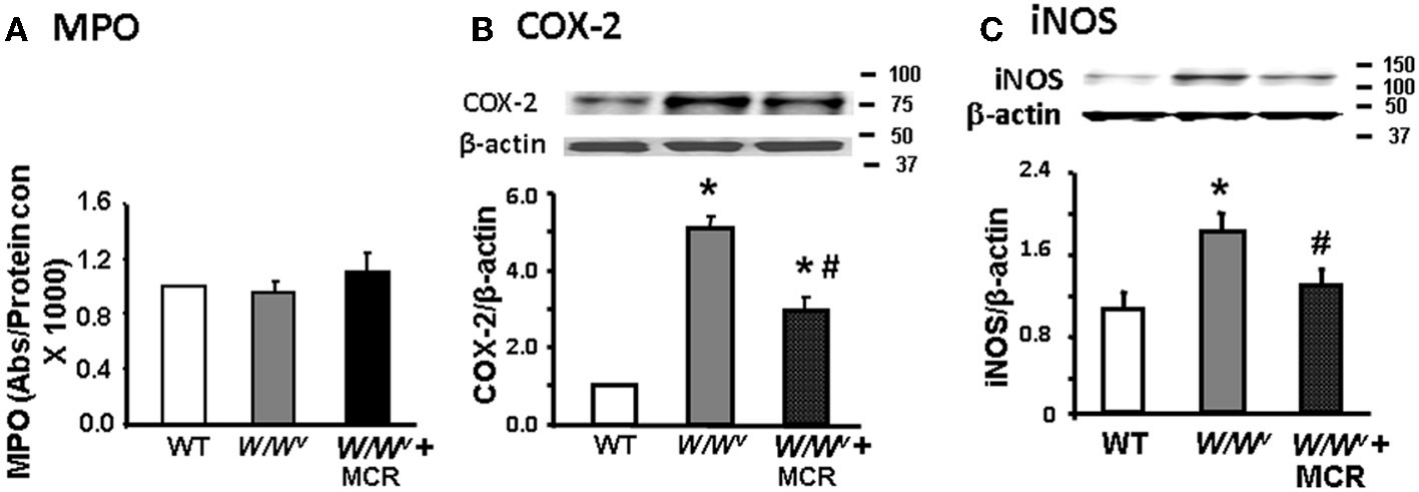
**Expression of inflammatory mediators in fundus muscularis externa from wild type, *W/W^v^*, and *W/W^v^* + mast cell reconstituted mice. (A)** Bar graph showing no significant difference in MPO activity between the three types of mice. **(B)** Western blot showing a significant increase in COX-2 expression in *W/W^v^* mice that was significantly reduced by mast cell reconstitution. **(C)** Western blot showing a significant increase in iNOS expression in *W/W^v^* mice that was significantly reduced by mast cell reconstitution, *n* = 5, ^*^p < 0.05 vs. wild type mice; ^#^*p* < 0.05 vs. *W/W^v^* mice.

The above findings indicated that impairment of fundus smooth muscle reactivity to Ach was not due to the depletion of ICC in *W/W^v^* mice. Therefore, we investigated whether the depletion of mast cells in *W/W^v^* mice created an inflammatory environment that impaired smooth muscle contractility. We found no significant difference in myeloperoxidase activity (MPO), a marker of accumulation of polymorphonuclear leukocytes, (Figure 3A) or H_2_O_2_ (data not shown) in the fundus muscularis externae of wild type, *W/W^v^* and *W/W^v^* mice after mast cell reconstitution. However, COX-2 and iNOS expression was significantly greater in the fundus of *W/W^v^* mice vs. the wild type mice (Figures 3B,C). Mast cell reconstitution partially but significantly, reversed the increase of COX-2 expression and completely reversed the increase of iNOS in *W/W^v^* mice (Figures 3B,C).

### Effects of COX-2 and iNOS inhibition on smooth muscle reactivity to Ach

#### Celecoxib

**Figure 4:**
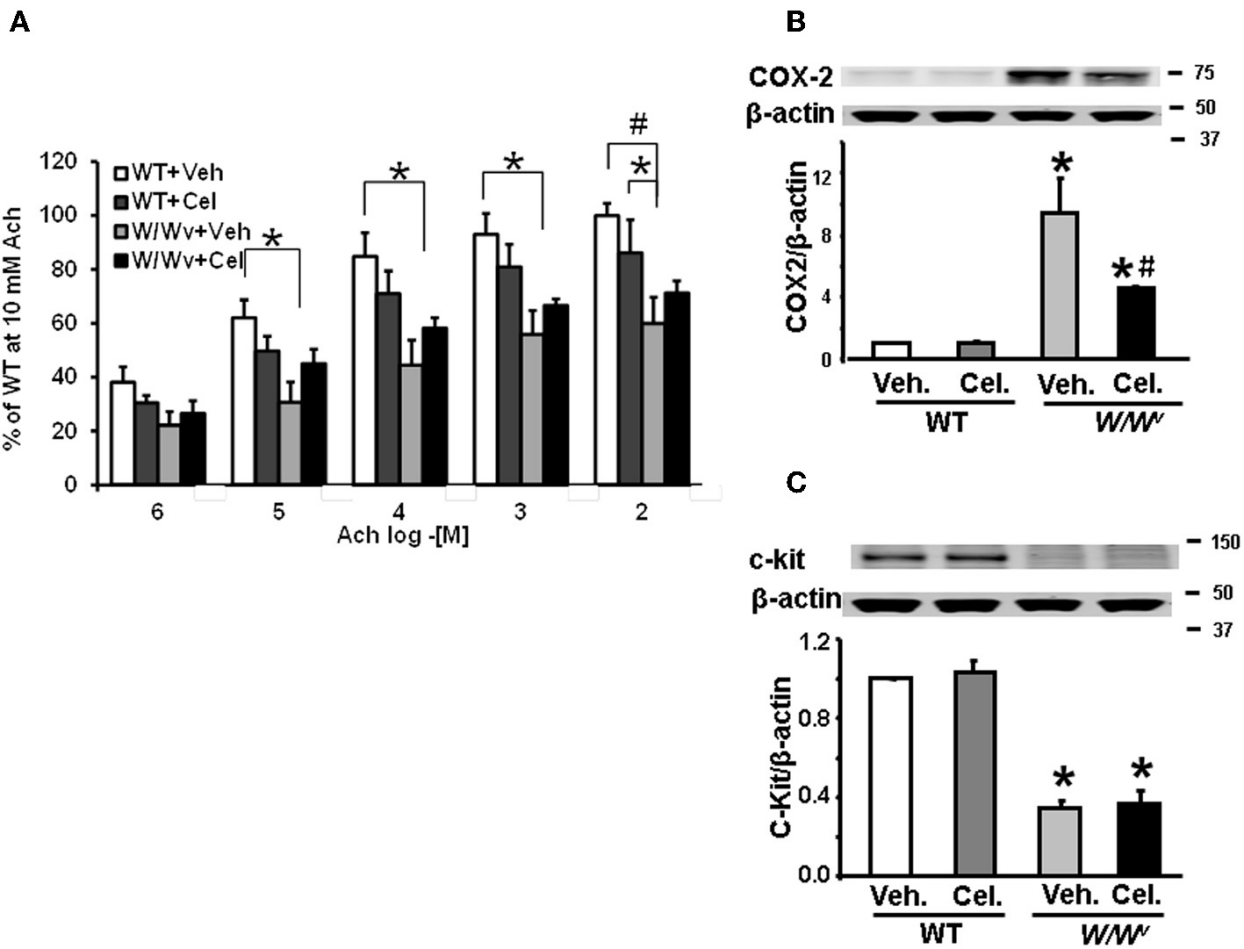
**Effects of daily i.p. administration of COX-2 inhibitor, celecoxib, for 3 days on fundus smooth muscle reactivity to Ach and c-kit expression. (A)** Smooth muscle reactivity to Ach was suppressed in *W/W^v^* mice compared with wild type mice. Celecoxib treatment partially restored smooth muscle reactivity to Ach, but it did not reach significance. **(B)** *W^v^* mutation significantly increased the expression of COX-2 in *W/W^v^* mice; celecoxib partially, but significantly reversed the elevation of COX-2. **(C)** *W^v^* mutation suppressed c-kit expression in *W/W^v^* vs. wild typemice. Celecoxib treatment did not affect c-kit expression in wild type or restore it in *W/W^v^* mice. *N* = 5–6 mice ^*^p < 0.05 vs. wild type mice. #*p* < 0.05 vs. WT + Cel.

To determine whether inhibition of COX-2 restored impaired fundus muscle response to acetylcholine in *W/W^v^* mice, we treated *W/W^v^*, and WT mice with celecoxib (10 mg/kg, i.p. once a day for 3 days) or vehicle (same volume, once daily for 3 days i.p.). Analysis of muscle bath data revealed significant main effects of genotype [*F*_1, 80_ = 20.7, *p* < 0.001] and acetylcholine concentration [*F*_1, 80_ = 38.6, *p* < 0.001]. Celecoxib treatment alone partially reversed the suppression of smooth muscle reactivity to Ach, but it failed to reach statistical significance [*F*_1, 80_ = 3.29, *p* = 0.076] (Figure 4A). No significant interaction between drug treatment and genotype was detected. Celecoxib treatment in wild type mice had no significant effect on smooth muscle reactivity to Ach. As shown previously, acetylcholine sensitivity in *W/W^v^* muscle strips was significantly less than in wild type strips and acetylcholine produced a dose dependent increase in the contractile response in muscle strips from all treatment groups. Daily i.p. administration of 10 mg/kg celecoxib for 3 days significantly suppressed the expression of COX-2 (Figure 4B), but had no effect on the reduced expression of c-kit in *W/W^v^* mice (Figure 4C).

#### L-NIL and L-NIL + celecoxib

**Figure 5:**
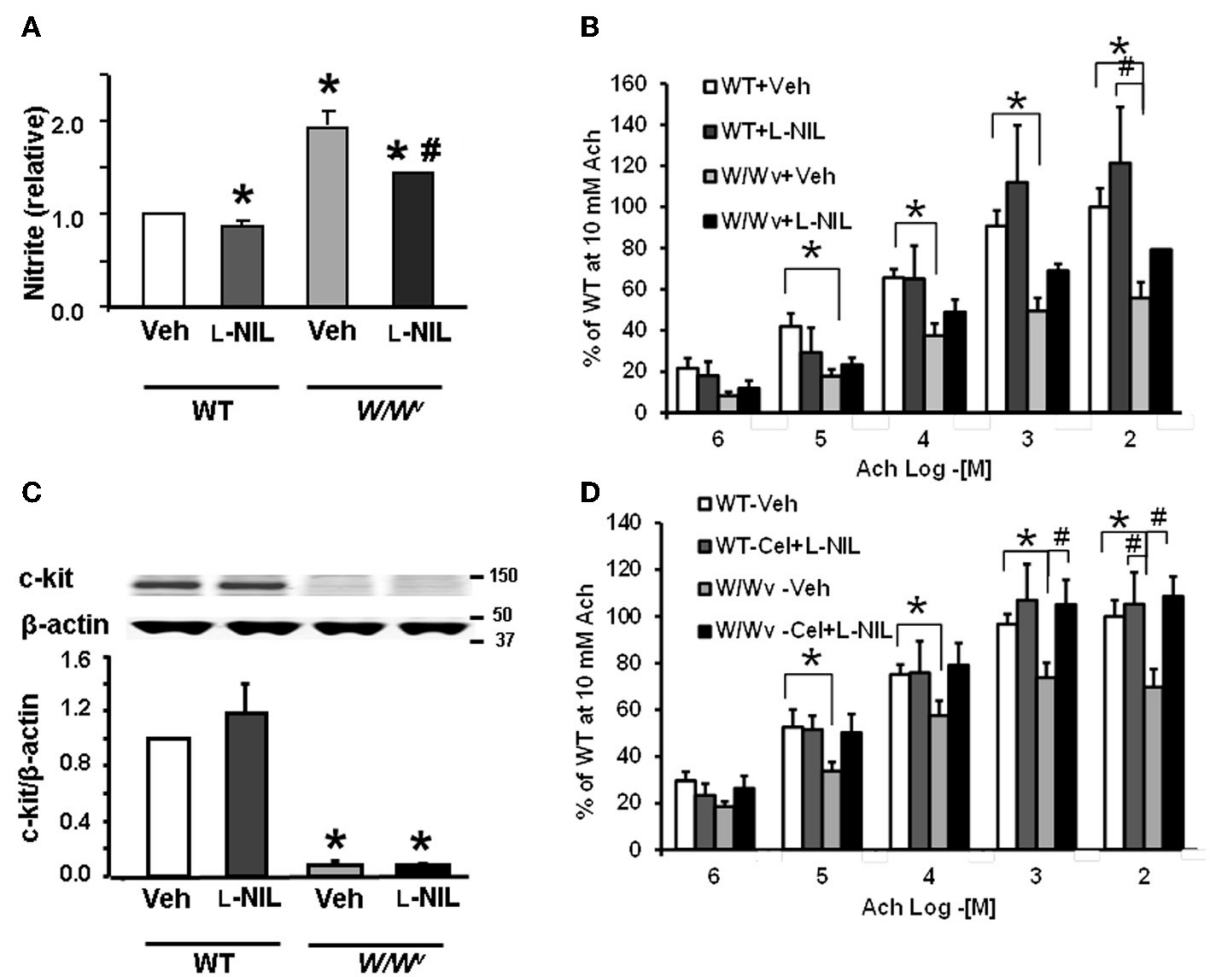
**Effect of i.p. treatment with L-NIL or L-NIL + celecoxib on fundus nitrite levels, smooth muscle reactivity to Ach and c-kit expression. (A)** Bar graph showing the effects of L-NIL treatment for 3 days on the average nitrite levels relative to that in Wt vehicle mice. Nitrite levels were significantly elevated in *W/W^v^* mice compared to Wt but were significantly reduced in *W/W^v^* and wild type mice by L-NIL treatment (^*^p < 0.05 vs. wild type + vehicle; ^#^*p* < 0.05 vs. *W/W^v^* + vehicle). **(B)** Bar graph showing the effects of L-NIL treatment on fundus muscle sensitivity to acetylcholine (Ach). The reactivity to Ach was suppressed in *W/W^v^* vs. the wild-type mice. Average contractility of strips from L-NIL treated *W/W^v^* mice was consistently greater than those from vehicle treated mice at all Ach concentrations tested, but these differences did not reach statistical significance (^*^p < 0.05 wild type + vehicle vs. *W/W^v^* + vehicle; ^#^*p* < 0.05 Wt + L-NIL vs. *W/W^v^* + vehicle). **(C)** C-kit expression was not increased by L-NIL treatment in *W/W^v^* mice. **(D)** Three-day treatment of *W/W^v^* mice with a combination of celecoxib and L-NIL completely reversed the suppression of smooth muscle reactivity to Ach in *W/W^v^* mice. *N* = 4 rats in each group. ^*^P < 0.05 vs. wild-type mice (WT) vehicle; ^#^*P* < 0.05 vs. *W/W^v^* vehicle mice.

Daily i.p. administration of iNOS inhibitor, N-iminoethyl-L-lysine (L-NIL; 5 mg/kg for 3 days) suppressed nitrite level in the fundus muscularis externa of the wild-type and *W/W^v^* mice, but had no significant effect on the reduced expression of c-kit (Figures 5A,B). The downregulation of nitrite level in the muscularis externa confirmed the efficacy of L-NIL dose. Analysis of muscle bath data revealed significant main effects of genotype [*F*_(1, 115)_ = 16.7, *p* < 0.001], acetylcholine concentration [*F*_(1, 115)_ = 52.6, *p* < 0.001] and a genotype × acetylcholine interaction [*F*_(1, 115)_ = 4.54, *p* = 0.038] (Figure 5C). L-NIL treatment alone partially reversed the suppression of smooth muscle reactivity to Ach in *W/W^v^* mice but it did not reach statistical significance [*F*_(1, 115)_ = 3.42, *p* = 0.071]; it had no effect in wild type mice.

Three-day treatment of *W/W^v^* mice with a combination of celecoxib and L-NIL, as described above, completely reversed the suppression of smooth muscle reactivity to Ach in *W/W^v^* mice (Figure 5D). Analysis of muscle bath data revealed significant main effects of genotype [*F*_(1, 150)_ = 10.0, *p* = 0.002], acetylcholine concentration [*F*_(1, 150)_ = 12.4, *p* = 0.001] and drug treatment × genotype interaction [*F*_(1, 150)_ = 21.6, *p* < 0.001]. This interaction together with *post-hoc* analysis (Figure 5D) showed that drug treatment significantly increased the sensitivity to acetylcholine of muscle strips from *W/W^v^* mice but had no significant effect on acetylcholine sensitivity of muscle strips from WT mice. As shown previously, acetylcholine sensitivity in *W/W^v^* muscle strips was significantly less compared to that in WT mice and acetylcholine produced a dose dependent increase in the contractile response in muscle strips from all treatment groups. These data demonstrated that concurrent inhibition of COX-2 and iNOS restored acetylcholine sensitivity in muscle strips from *W/W^v^* mice.

### Effects of mast cell reconstitution, sulforaphane and L-NIL + celecoxib treatments on nNOS and chat expression in wild type and *W/W^v^* mice

**Figure 6:**
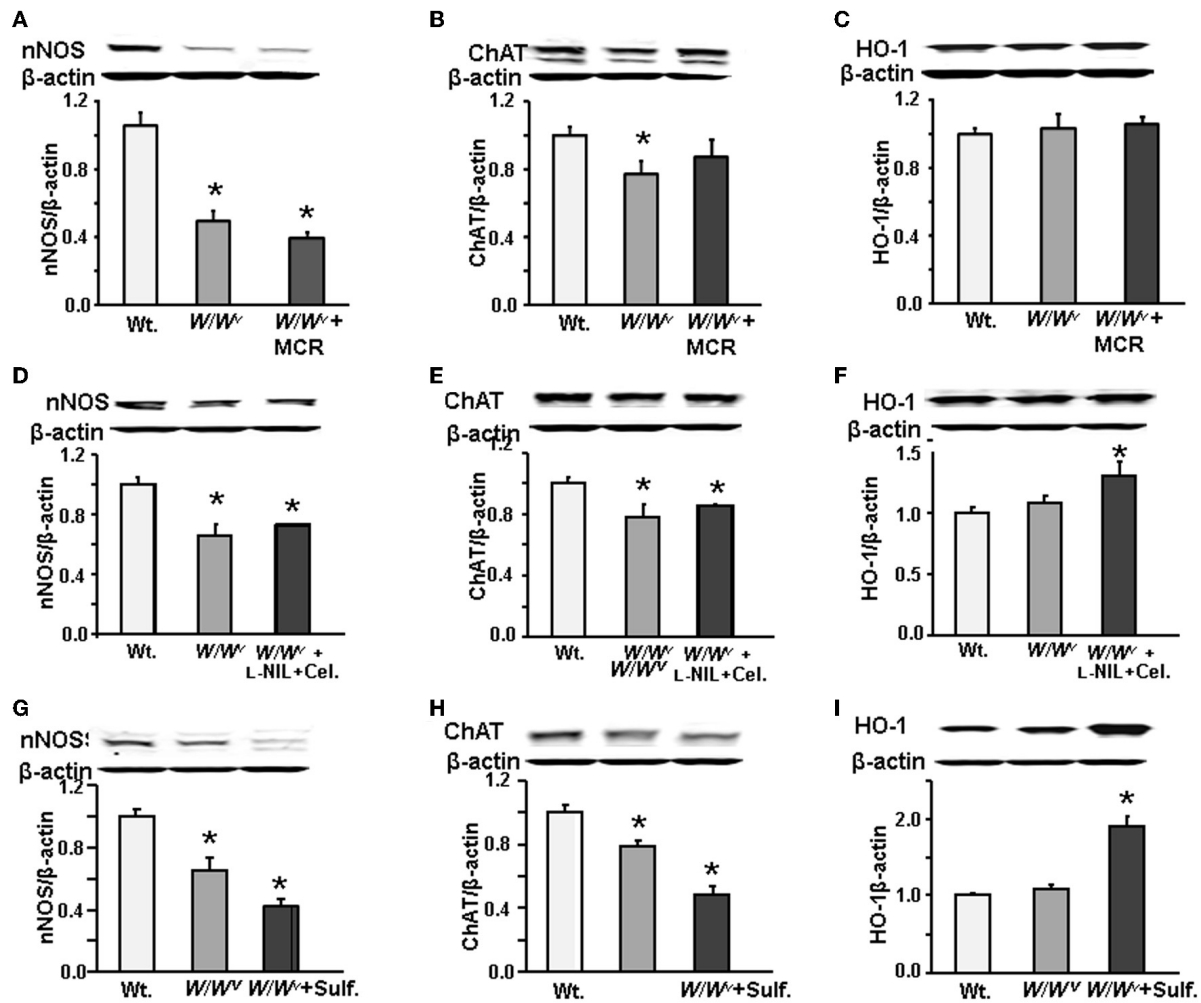
**Western blots showing nNOS, ChAT and HO-1 expression in WT, *W/W^v^*, and *W/W^v^ + MCR mice*. (A)** nNOS expression decreased in *W/W^v^* vs. wild type (WT) mice, mast cell reconstitution did not restore it. **(B)** ChAT expression decreased *in W/W^v^* mice, mast cell reconstitution restored it partially. **(C)** There was no significant difference in HO-1 expression between WT and *W/W^v^* mice. Mast cell reconstitution in *W/W^v^* mice had no effect on heme oxygenase (HO-1) expression. **(D)**, **(E)**, and **(F)** Combined L-NIL and celecoxib (Cel.) had no effect on the down regulation of nNOS or ChAT, but they upregulated the expression of HO-1 in *W/W^v^* mice. **(G)**, **(H)**, and **(I)** 3-day treatment with antioxidant sulforaphane had no effect of the downregulation of nNOS or ChAT, but it upregulated HO-1 expression in *W/W^v^*mice. *N* = 5 mice in each group. ^*^p < 0.05 vs. wild type mice. Each protein appeared as a single band corresponding to the expected molecular weight. Molecular weight was calculated from migration relative to protein standards on the gel. nNOS (155 kd) was next to 150 kDa standard, ChAT (70 kd) migrated between 75 kDa standard and HO-1 (32 kDa) migrated between the 37 kDa and 25 kDa standards.

Next, we investigated whether the expressions of enzymes nNOS and ChAT that respectively regulate the synthesis of NO and Ach are altered in *W/W^v^* mice. We found that both **nNOS** and ChAT expression in the gastric fundus muscularis externa of *W/W^v^* mice were suppressed vs. the wild type mice (Figure 6A). Mast cell reconstitution did not restore nNOS (Figure 6A) expression; however, it marginally restored ChAT expression (Figure 6B). Heme oxygenase-1 (HO-1) expression in *W/W^v^* mice was not different from wild type mice and mast cell restoration had no effect on its expression (Figure 6C). Additional experiments in a separate group of *W/W^v^* mice showed that combined celecoxib and L-NIL treatment also did not restore nNOS (Figure 6D) or ChAT (Figure 6E) expression, even though it upregulated the expression of HO-1 (Figure 6F). We then tested whether treatment of *W/W^v^* mice with the antioxidant sulforaphane [1-isothiocyanato-(4R,S)-(methylsulfinyl) butane] (5 mg/kg daily for 3 days, i.p.) restores nNOS (Figure 6G) or ChAT expression (Figure 6H). Sulforaphane treatment did not restore the expression of nNOS (Figure 6G) or ChAT (Figure 6H), even though it significantly increased the expression of HO-1 (Figure 6I), as expected.

## Discussion

Our findings show that use of the *W/W^v^* mouse model to investigate the role of ICC in regulating smooth muscle and enteric neural function is more complex than originally thought. The initial assumption was that the noted alterations in smooth muscle phenotype and enteric neurotransmission in *W/W^v^* mice were due to the loss of ICC. However, our findings show that the concurrent loss of mast cells in *W/W^v^*mice creates an inflammatory environment in the fundus muscularis externa that impairs smooth muscle reactivity to Ach and down regulates the expression of ChAT and nNOS that respectively generate Ach and NO. These complications would severely limit use of the *W/W^v^* mice in investigating the role of ICC in mediating enteric neuronal input to smooth muscle cells. Mast cell distribution is heterogeneous in the gastrointestinal track (Mikkelsen, 2010). One study found that amplitude of the jejunum longitudinal muscle contraction to 10^−6^ M carbachol did not differ between the control, *W/W^v^* and mast cell reconstituted *W/W^v^* mice (Vallance et al., 2001). The type of inflammation induced by mast cell depletion in other parts of the gastrointestinal tract in *W/W^v^* mice and its effects on circular smooth muscle contractility and expression of ChAT and nNOS remain unknown.

Previous studies in *W/W^v^* and Sl/Sl^d^mice used only low concentrations of 10^−8^ M to 10^−5^ M to evaluate smooth muscle contractility in response to Ach (Ward et al., 2000; Beckett et al., 2002). We confirm their findings that at low concentration (e.g., 10^−6^ M) the reactivity to cholinergic agonists does not differ between the *W/W^v^* and wild type mice. However, in our study, differences between smooth muscle reactivity to Ach between the wild-type and *W/W^v^*mice began to appear at concentration of 10^−4^ M. Ach acts on muscarinic receptors on visceral smooth muscle cells in the range used by us (10^−6^ M to 10^−2^ M) (Chen et al., 1995; Hegde and Eglen, 1999; Sellers et al., 2000) and we found the concentration-response curve to show classical steep gradient at lower concentrations and moderate gradient at higher concentrations, without reaching saturation. We believe the use of only low concentrations of cholinergic agonists might have led others to conclude lack of alterations in smooth muscle contractility in *W/W^v^* mice (Ward et al., 2000; Beckett et al., 2002).

Our findings provide critical new evidence in the controversy about the putative role of ICC acting as the mediators of neuronal input to smooth muscle cells. The initial approach to assess the role of ICC-IM in mediating cholinergic enteric neuronal input to smooth muscle cells in the fundus was to compare the amplitudes of excitatory junction potentials (EJPs) in response to electrical field stimulation between the wild-type and W*/W^v^* mice (Ward et al., 2000). The EJPs acted as surrogates for smooth muscle contraction. The investigators found that EJP amplitude was greatly reduced in *W/W^v^* vs. the wild-type mice. They reported, in addition, that the smooth muscle cells generated similar electrical and mechanical response to exogenous Ach, the density of cholinergic neurons did not differ and ^14^[C]choline release by EFS was similar in wild type and *W/W^v^* mice, suggesting that the smooth muscle contractility and cholinergic nerve function were normal in W*/W^v^* mice. Therefore, the conclusion was drawn that the absence of ICC-IM in the fundus caused near obliteration of the EJP; therefore, the ICC-M mediated cholinergic neuronal input to the smooth muscle cells.

By contrast, our findings show that smooth muscle reactivity to Ach is suppressed in *W/W^v^* mice. We identified the cause of suppression of smooth muscle contractility as the well-known concurrent depletion of mast cells in *W/W^v^* mice (Wershil and Galli, 1994). The decrease in smooth muscle contractility was independent of the decrease in c-kit and disruption of ICC networks; mast cell reconstitution completely restored smooth muscle reactivity to Ach without affecting the suppression c-kit and disruption of ICC networks. It is, therefore, likely that the decrease in amplitude of the EJPs in response to EFS in *W/W^v^* mice was at least in part due to the suppression of smooth muscle reactivity to Ach, rather than due to the absence of ICC. Other studies comparing alterations of smooth muscle and nitrergic inhibitory functions in nNOS^−/−^ and *W/W^v^* mice also arrived at the conclusion that the smooth muscle response to EFS is impaired in other parts of the gut in *W/W^v^* mice (Sivarao et al., 2001, 2008; Alberti et al., 2007; Farre et al., 2007; Huizinga et al., 2008; Zhang et al., 2010).

Fundus does not appear to be the only area to show smooth muscle abnormalities in *W/W^v^* mice. Other investigators found hypotension, hyperpolarization of resting membrane potential and reduction in spontaneous unitary potentials in pyloric and LES sphincters (Sivarao et al., 2001, 2008; Zhang et al., 2010). The underlying mechanisms of these alterations, specifically, the role of mast cell depletion in these tissues, remain unknown. However, previous studies show that several regulatory proteins and ion channels in smooth muscle cells are targets for modulation of their expression by inflammatory mediators (Collins et al., 1989; Lu et al., 1999; Akbarali et al., 2000; Liu et al., 2001; Kinoshita et al., 2003; Cong et al., 2007; Chandrasekharan et al., 2011; Shi et al., 2011b). Taken together, various types of smooth muscle dysfunction in *W/W^v^* mice may occur independent of the loss of ICC; therefore, monitoring smooth muscle contractile response to electrical field stimulation (EFS) in *W/W^v^* mice (Burns et al., 1996; Ward et al., 1998, 2000, 2004, 2006) to test the intermediary role of ICC in neurotransmission can be misleading.

One study found that *in vitro* atropine-sensitive force generation in the fundus circular muscle strips in response to EFS or carbachol was the same or higher in *Ws/Ws* vs. the wild type rats (Zhang et al., 2011). On the other hand, the *ex vivo* atropine-sensitive generation of fundus pressure in response to distension was lower in *Ws/Ws* vs. wild type rats. The reasons for the suppression of smooth muscle contractility in circular muscle strips from the *W/W^v^* mice, but not from the *Ws/Ws* rats that also lack the ICC-IM remain unclear.

ChAT and nNOS expression were also suppressed in the fundus muscularis externa. However, the mechanisms mediating the suppression of these enzymes remain unknown; mast cell reconstitution, COX-2 and iNOS inhibitors or suppressors of oxidative stress did not reverse the suppression of ChAT or nNOS. Complete depletion of nNOS in nNOS^−/−^ mice obliterates smooth muscle relaxation to EFS. In our study, the ChAT and nNOS decreased about 30 and 50% respectively in the fundus of W/W^v^ vs. wild-type mice. The precise effects of these reductions on the role of gastric fundus in regulating gastric emptying remains unknown. Other investigators found that a reduction of 11–70% in the expression of these enzymes, measured by western blotting or reduction in the number of neurons, induced alterations in *in vitro* and *in vivo* motility function. The potential roles of partial deficits in nNOS and ChAT expression should be considered in evaluating the putative role of ICC as mediators of enteric neurotransmission in the gut.

A multitude of inflammatory molecules, including cytokines, chemokines, nitric oxide, oxygen free radicals, growth factors, and arachidonic acid metabolites can mediate the adverse effects of inflammation on biological tissues. We found that mast cell depletion in the gastric fundus of *W/W^v^* mice elevates iNOS and COX-2, both of which together, but not individually, contributed to the suppression of smooth muscle reactivity to Ach. Mast cell reconstitution or the concurrent inhibition of both mediators completely reversed the suppression of smooth muscle reactivity to Ach.

Preclinical models showed that inflammation in partial gut obstruction (Chang et al., 2001) and intestinal handling during surgery impairs smooth muscle function and reduces ICC volume by transforming them to myofibroblast-like cells (Kalff et al., 2000; Schwarz et al., 2001; Yanagida et al., 2007). Both procedures induced COX-2 and iNOS expression (Kalff et al., 2000; Schwarz et al., 2001; Yanagida et al., 2007; Shi et al., 2011a). However, on removal of partial obstruction, the ICC volume recovered earlier than the smooth muscle function, suggesting that the ICC might not have caused smooth muscle dysfunction. Similar disconnects were noted during the recovery of ICC volume and smooth muscle function in TNBS-induced inflammation (Kiyosue et al., 2006). The inhibitors of iNOS and COX-2 blocked the decrease in ICC volume as well as smooth muscle dysfunction induced by intestinal handling. Together, these findings suggest that inflammation may impair smooth muscle function and decrease ICC volume independent of each other. The ICC volume in the *W/W^v^* mice did not recover after mast cell reconstitution or the blockade of COX-2 and iNOS because of the genetic mutation in these mice.

Clinical studies using immunohistochemical, morphological and ultrastructural approaches found that ICC were depleted and their networks disrupted in several human gut motility disorders, including diabetes mellitus (He et al., 2001; Nakahara et al., 2002; Iwasaki et al., 2006; Miller et al., 2008; Pasricha et al., 2008), slow transit constipation (He et al., 2000; Lyford et al., 2002), Hirschprung's disease (Vanderwinden et al., 1996), gastroparesis (Zarate et al., 2003), chronic constipation (Yu et al., 2002), and megacolon (Wedel et al., 2002). The depletion of ICC volume and impairment of ICC networks were proposed as the underlying causes of disordered motility function. However, a cause-and-effect relationship was not established between the loss of ICC and smooth muscle (slow waves or smooth muscle reactivity to Ach or NO) or enteric neuronal dysfunction in these studies. Our findings suggest an alternate possibility that the affected segment of the gut in these disorders develops an inflammatory response that impairs smooth muscle and enteric neuronal functions in parallel with the impairment of ICC networks and volume. Indeed. oxidative stress in diabetic human colon reduces the numbers of nNOS and ChAT immunoreactive neurons, resulting in suppression of relaxation and contraction of smooth muscle strips in response to EFS (Chandrasekharan et al., 2011), while alterations in prostaglandin and COX enzymes impair motility in slow transit constipation (Cong et al., 2007).

The development of gastroparesis in the nonobese diabetic mouse model illustrates the role of inflammation in delayed gastric emptying independent of loss of c-Kit expression. The gastric tissue in these mice developed oxidative stress, impaired nNOS expression and depletion of c-kit, leading to gastroparesis (Choi et al., 2008). These defects were resolved on treatment with hemin that upregulates heme oxygenase-1 (HO-1) and reduces oxidative stress. Since c-kit expression is not known to regulate nNOS expression, the oxidative stress likely suppressed nNOS expression independent of c-kit suppression. The parallel impairment of ICC volume and networks was likely collateral damage in diabetic gastroparesis.

In conclusion, the *W/W^v^* mouse appears to be a complex model to investigate the role of ICC in motility function. The concurrent loss of mast cells in these mice generates an inflammatory environment in the muscularis externa that can suppress smooth muscle contractility and the expression of ChAT and nNOS in myenteric neurons. The deficiency of ChAT and nNOS might contribute to impaired neuronal function. Mast cell deficiency in the fundus upregulated the inflammatory mediators, COX-2 and iNOS. Mast cell reconstitution or concurrent inhibition of COX-2 and iNOS restored smooth muscle contractility, independent of the loss of ICC. The effects of loss of mast cells on smooth muscle contractility and expression of ChAT and nNOS might be different in other parts of the gastrointestinal track due to the heterogeneous distribution of mast cells in the gut.

### Conflict of interest statement

The authors declare that the research was conducted in the absence of any commercial or financial relationships that could be construed as a potential conflict of interest.
